# Assessing statistical significance in causal graphs

**DOI:** 10.1186/1471-2105-13-35

**Published:** 2012-02-20

**Authors:** Leonid Chindelevitch, Po-Ru Loh, Ahmed Enayetallah, Bonnie Berger, Daniel Ziemek

**Affiliations:** 1 Computational Sciences Center of Emphasis, Pfizer Worldwide Research & Development, Cambridge, MA, USA; 2 Mathematics Department and Computer Science and Artificial Intelligence Laboratory, Massachusetts Institute of Technology, Cambridge, MA, USA; 3 Compound Safety Prediction, Pfizer Worldwide Research & Development, Cambridge, MA, USA

## Abstract

**Background:**

Causal graphs are an increasingly popular tool for the analysis of biological datasets. In particular, signed causal graphs--directed graphs whose edges additionally have a sign denoting upregulation or downregulation--can be used to model regulatory networks within a cell. Such models allow prediction of downstream effects of regulation of biological entities; conversely, they also enable inference of causative agents behind observed expression changes. However, due to their complex nature, signed causal graph models present special challenges with respect to assessing statistical significance. In this paper we frame and solve two fundamental computational problems that arise in practice when computing appropriate null distributions for hypothesis testing.

**Results:**

First, we show how to compute a p-value for agreement between observed and model-predicted classifications of gene transcripts as upregulated, downregulated, or neither. Specifically, how likely are the classifications to agree to the same extent under the null distribution of the observed classification being randomized? This problem, which we call "Ternary Dot Product Distribution" owing to its mathematical form, can be viewed as a generalization of Fisher's exact test to ternary variables. We present two computationally efficient algorithms for computing the Ternary Dot Product Distribution and investigate its combinatorial structure analytically and numerically to establish computational complexity bounds.

Second, we develop an algorithm for efficiently performing random sampling of causal graphs. This enables p-value computation under a different, equally important null distribution obtained by randomizing the graph topology but keeping fixed its basic structure: connectedness and the positive and negative in- and out-degrees of each vertex. We provide an algorithm for sampling a graph from this distribution uniformly at random. We also highlight theoretical challenges unique to signed causal graphs; previous work on graph randomization has studied undirected graphs and directed but unsigned graphs.

**Conclusion:**

We present algorithmic solutions to two statistical significance questions necessary to apply the causal graph methodology, a powerful tool for biological network analysis. The algorithms we present are both fast and provably correct. Our work may be of independent interest in non-biological contexts as well, as it generalizes mathematical results that have been studied extensively in other fields.

## Background

Causal graphs are a convenient representation of causal relationships between variables in a complex system: variables are represented by nodes in the graph and relationships by directed edges. In many applications the edges are also signed, with the sign indicating whether a change in the causal variable positively or negatively affects the second variable. Causal graphs can serve as predictive models, and conclusions can be drawn from comparing the models' predictions to experimental measurements of these variables. Pollard et al. [1] pioneered the use of large-scale causal graphs to interpret gene expression data and the approach has been used successfully in several contexts [2-4]. We present our own causal reasoning approach in our companion paper [5]; here we give a brief overview.

**Figure 1:**
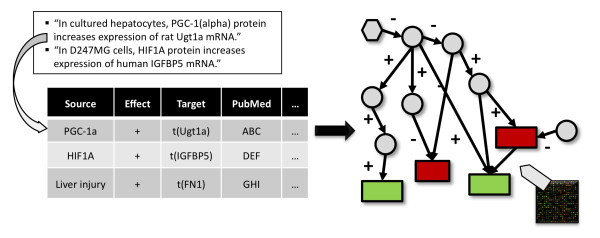
**Illustration of the causal graph methodology**. Schematic depiction of a set of relationships curated from the literature and transformed into a causal graph, used to explain gene expression data.

Published research in biology provides a wealth of regulatory relationships within the cell that we mine to produce a causal network. The edges in this network are directed (by the flow of causality among the corresponding variables) and signed (by the sign of the correlation between the variables). Directed paths within the network thus predict putative upregulation and downregulation that would be effected downstream by changes in the level of a given entity (i.e., vertex in the graph). Our companion paper [5] shows that this reasoning can be applied to the inverse problem: given data from a gene expression assay, our causal network enables us to infer potential upstream causes for the measured gene expression changes. The key output of the method is a list of upstream hypotheses that explain a large fraction of the observed changes in a statistically significant manner. As hypotheses are based on existing literature, they are easily interpretable by biological experts and can provide building blocks for a more comprehensive understanding of causal drivers of the processes under consideration. Figure 1 provides a schematic of the approach.

In this paper, we study the problem of evaluating statistical significance of the conclusions drawn from a causal graph-based model given a particular gene expression dataset. To form a null distribution, either the correspondence between gene transcripts and experimental expression values or the connectivity of the graph can be randomized. Thus, the statistical significance question splits into two subproblems. First, how likely is it for the same level of agreement between predicted and observed regulation to be achieved when the classification of gene transcripts (as upregulated, downregulated, or neither) is randomly drawn from a family of all classifications with similar characteristics? Second, how likely is it to occur when the causal graph is randomly drawn from a family of all causal graphs with similar characteristics?

Answering the first question amounts to computing the distribution of the dot product of two vectors with components in {-1, 0, 1}, each drawn randomly from the family containing all such vectors with a fixed number of components of each value. This problem, which we call Ternary Dot Product Distribution, generalizes Fisher's exact test [6] to ternary variables and we thus believe it is of independent interest. Fisher's exact test is ubiquitously used in gene set enrichment analysis and many other areas of computational biology [7]. This test is appropriate to assess statistical significance of enrichment in many settings but neglects the sign of differential regulation. In many cases, the sign of the regulation is available and could be harnessed to obtain additional insights. One example where our proposed extension is directly applicable is as an alternative scoring mechanism for the well-known Connectivity Map approach [8].

Answering the second statistical significance question analytically does not appear to be possible, but the desired likelihood may be approximated by sampling uniformly at random from the family of all causal graphs with the same basic structure as the original causal graph: namely, the same positive and negative in- and out-degrees of each vertex. Because of the structure of the problem, even drawing one causal graph from this family is challenging. We call this the Causal Graph Randomization problem. Previous work on the problem of graph randomization has focused on undirected graphs [9-11]; the context of directed graphs is less well-studied theoretically [12-17] despite finding many uses in bioinformatics [18-20].

The rest of this paper is organized as follows. We begin by describing the regulatory network model based on causal graphs and discuss the way conclusions are drawn from it and the importance and subtleties of computing their statistical significance. We then describe the Ternary Dot Product Distribution problem and present two efficient algorithms to solve it: an algorithm with complexity cubic in the number of variables (i.e., vertices) in the graph but requiring computation in exact arithmetic, and an algorithm with a weaker complexity guarantee but numerically stable and efficient in practice. Finally, we discuss the challenges of the Causal Graph Randomization problem and present a practical algorithm for it using local graph operations, and conclude by describing future work.

### Model Description

The two fundamental properties of causal relationships between biological entities are (1) the direction of causality between them; and (2) the qualitative response (i.e., upregulation or downregulation) of the second entity when the first one is upregulated or downregulated. This information can be encapsulated in a signed directed graph *G *= (*V, E*) whose nodes *V *are genes, transcripts, compounds, or biological processes, and where a directed edge from node *a *to node *b *means that the abundance or activity of *b *is regulated by the abundance of *a*. The edge (*a, b*) is labeled with a "+" sign if the regulation is positive (i.e., an increase in *a *leads to an increase in *b*), and it is labeled with a "-" sign if the regulation is negative. We call *G *a *causal graph*.

For any two nodes *a *and *z *not necessarily connected by an edge, the causal graph *G *models the effects of a change in the abundance of *a *on the abundance of *z *by tracing the shortest directed path from *a *to *z *in *G *and then evaluating its sign, given by the product of the signs of the edges along the path. If this overall sign turns out to be a plus sign, it is expected that *a *upregulates *z*, and if it is a minus sign, that *a *downregulates *z *[1].

#### Hypothesis scoring

Given a gene expression dataset, we may classify gene transcripts into three families: significantly upregulated, significantly downregulated, and not significantly regulated. We refer to this classification as the *experimental classification*. We wish to understand what perturbations may have led to these observations.

Given a particular entity *v *∈ *V *in our causal graph, we can examine the predicted effects of upregulating or downregulating it. We call *v *together with the direction of perturbation a *hypothesis*. This hypothesis also classifies the gene transcript nodes in the graph into three families: those predicted to be upregulated by the perturbation of *v*, those predicted to be downregulated by the perturbation of *v*, and those not predicted to be regulated by *v*. We refer to this classification as the *predicted classification*.

In order to evaluate the goodness-of-fit of a particular hypothesis to the observed gene expression dataset, we declare a prediction to be *correct *if the predicted sign matches the experimental sign and the regulation was significant: i.e., both signs are + or both are -. In case of a mismatch (a + and a -), we declare the prediction to be *incorrect*. In all other cases, we declare the prediction to be *ambiguous*. We may now score a hypothesis by awarding 1 point for each correct prediction, -1 for each incorrect prediction, and 0 for each ambiguous prediction.

#### Statistical significance

The scores computed for each putative hypothesis provide us with an overall ranking of all hypotheses. However, a good score does not necessarily imply good explanatory power, because of possible connectivity differences between the transcript nodes of *G*. In particular, "hubs" with high degree are more likely to have higher scores regardless of which genes are experimentally observed to be significantly regulated. Therefore, we also need to look at the statistical significance of each score when the gene expression data is randomized, preserving the number of upregulated and downregulated gene transcript nodes, but not the nodes themselves.

In addition, we need to understand how significant the rank of a hypothesis is with respect to another null model, in which the gene expression data remains fixed but the causal graph is allowed to vary, only keeping basic connectivity properties. More specifically, we examine the rank of a hypothesis of interest in the family of graphs with the same sequence of positive and negative in-degrees and out-degrees as *G*, but randomly connected otherwise. If these degrees rather than the full structure of *G *suffice to give a hypothesis of interest a good rank, this hypothesis should not be deemed statistically significant.

### Illustrative Example

To build intuition for the proposed method we outline an example application based on previously published experimental data (GEO accession GSE7683 [21]) and a large-scale causal network containing approximately 250,000 unique relationships licensed from Ingenuity, Inc. and Selventa, Inc. The original study was devised to study the effect of dexamethasone on the differentiation and development of primary mouse chondrocytes using gene expression microarrays. Interestingly, the authors report difficulties in drawing clear conclusions about the pathways and biological categories affected by dexamethasone using traditional microarray analysis methods and Gene Ontology annotations. The authors suggest that the difficulty may be due to modest response to dexamethasone (i.e., weak signal compared to background noise) that limited the ability of traditional approaches to make inference [21].

**Figure 2:**
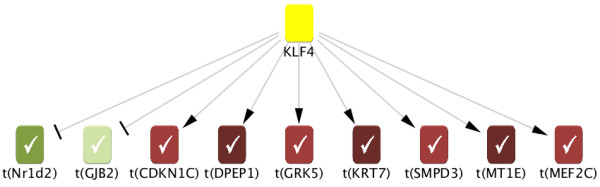
**Scoring of an example hypothesis**. Illustration of scoring for the *KLF4+ *hypothesis based on the experimental dataset discussed in the main text. Arrows illustrate predicted upregulation or downregulation of all experimentally regulated transcripts one step downstream of KLF4. In this case, all predictions match with experimental observations, resulting in 9 correct and 0 incorrect predictions and a corresponding score of 9.

Our approach provides a statistical framework for causal inference that may be particularly valuable in such a situation. As outlined above, we consider each entity in our causal graph together with a direction of perturbation as a hypothesis; based on the network model, perturbing the entity should effect changes downstream, and we assess significance of the concordance between the predicted and experimentally measured changes by computing p-values based on the Ternary Dot Product and Causal Graph randomized null models. For simplicity, in this example we only consider predicted downstream effects one step downstream of each entity. Figure 2 illustrates the scoring for one particular hypothesis, *KLF4+ *(i.e., upregulation of KLF4). Note that graph entities are not limited to genes or transcripts but may include more abstract concepts tied to expression changes in the literature; an example we will encounter below is *Response to hypoxia*. In this case, the "direction of perturbation" included in a hypothesis is also to be understood more abstractly: e.g., *Response to hypoxia+ *corresponds to an increase in the effects of hypoxia (as opposed to a concrete "upregulation").

Table 1 shows the top ten hypotheses obtained from the dexamethasone treatment data (specifically, the 24 hr time point) along with corresponding computed p-values. Five of the top hypotheses directly reflect the primary experimental perturbation: the perturbation itself (*Dexamethasone+*), the target receptor (*NR3C1+*), its drug family (*Glucocorticoid+*) and two other glucocorticoids (*Hydrocortisone+ *and *Triamcinolone acetonide+*). Other top hypotheses describe major players in chondrocyte development and differentiation. For example, *Response to hypoxia+ *may reflect the central role of hypoxia response factors in the development and survival of avascular tissues such as the chondrocytes being studied here [22]. In fact, examination of the biological context of the evidence supporting *Response to hypoxia+ *revealed corresponding results in the literature such as the promotion of chondrocyte differentiation by hypoxia [23]. Similarly, KLF4 (shown with supporting transcriptional evidence in Figure 2) is an important gene in cell differentiation and chondrogenesis [24] and has been shown to be upregulated during hypoxia-induced mesenchymal stem cell differentiation [25].

**Table 1:** Top hypotheses by score and corresponding p-values on an example dataset

Rank	Hypothesis Name	Correct	Incorrect	Score	Ternary Dot Product *p*	Causal Graph *p*
1	Response to Hypoxia+	48	9	37	2 × 10^-12^	< 0.001
2	Dexamethasone+	20	4	16	6 × 10^-6^	< 0.001
3	Hydrocortisone+	17	4	13	1 × 10^-8^	< 0.001
4	PGR+	12	1	11	6 × 10^-8^	< 0.001
5	SRF+	10	0	10	3 × 10^-5^	< 0.001
6	KLF4+	9	0	9	3 × 10^-6^	< 0.001
7	NR3C1+	12	4	8	7 × 10^-4^	< 0.001
7	Glucocorticoid+	12	4	8	8 × 10^-5^	< 0.001
7	CCND1+	9	1	8	3 × 10^-4^	< 0.001
7	Triamcinolone acetonide+	8	0	8	9 × 10^-7^	< 0.001
...	...	...	...	...	...	...
17	NRF2+	9	4	5	0.18	0.07

Top hypotheses by score in an example experimental dataset of dexamethasone-stimulated chondrocytes (GEO accession GSE7683 [21]). Each hypothesis is scored by the difference between the numbers of correct and incorrect predictions. Significance is assessed by the Ternary Dot Product and Causal Graph Randomization p-values discussed in the text; the latter numbers are estimates based on 1000 runs of graph randomization and for this reason are always a multiple of 0.001. When no randomized graph with a better score for the given hypothesis is detected, we indicate that as "p < 0.001." Note that hypotheses with the same numbers of correct and incorrect predictions do not necessarily have the same p-values because the significance calculation takes into account the full contingency table for each hypothesis; some hypotheses result in more predicted regulations than others.

Importantly, hypotheses are based on overlapping but different sets of regulated transcripts. Thus, while we assess significance of each hypothesis in isolation, the evidence shared among hypotheses should be helpful in building a more global understanding. For instance, 50% of the *KLF4+ *transcriptional evidence is also part of the *Response to hypoxia+ *evidence. This supports a major role of hypoxia in chondrogenesis which is partially mediated through KLF4.

Only 23 of the top 50 hypotheses by score pass a significance cutoff of 0.001 for both metrics, indicating the utility of significance assessment--not just score--in discerning hypotheses worthy of further investigation. For example, *NRF2+*, ranked 17th by score, is *not *deemed statistically significant according to our metrics; this is consistent with current knowledge as NRF2 negatively regulates chondrocyte differentiation contrary to the reported effect of dexamethasone. In contrast to our significance tests, a standard test for enrichment based on Fisher's Exact Test would have given a p-value < 10^-5^, a result that is probably spurious.

This example is not meant as a comprehensive discussion of the affected biology but should provide some intuition how the proposed measures can be used. For complex biological phenotypes, many hypotheses may be reported as significant that may include overlapping but distinct sets of transcriptional changes as supporting evidence. While our proposed metrics judge significance of single hypotheses independently, the results provide a statistically well-founded substrate on which to form a more comprehensive picture of potential drivers of the observed expression changes.

## Results

We divide this section into two parts corresponding to the two statistical significance questions we address: Ternary Dot Product Distribution and Causal Graph Randomization.

### Ternary Dot Product Distribution

We begin by establishing notation and phrasing the problem in a slightly more abstract setting which we find helpful for investigating its mathematical structure.

#### Problem definition

A *ternary classification *of a ground set T (such as the gene transcript nodes of the causal graph *G *in our motivating example) is a function from T to {-1, 0, 1}. Given an arbitrary but fixed ordering of the elements of T, we can naturally represent a ternary classification *C *of T as a ternary vector **u**(*C*) whose *i*-th component is the value of *C *on the *i*-th element of T. Then, for two ternary classifications *C *and *C' *of T, the *agreement *between *C *and *C' *(corresponding to the goodness-of-fit in our motivating example) is computed as the dot product **u**(*C*) · **u**(*C'*).

We are interested in understanding the distribution of the agreement between the fixed experimental classification *C *and a random classification whose parameters (numbers of -1, 0 and 1 components) are taken from the predicted classification *C'*. In other words, given two classifications *C *and *C' *of T, we are interested in the distribution of the agreement between *C *and a randomized version of *C' *over all possible randomizations, where a randomization of *C' *is a classification $C_{R}^{\prime }$ of T with the same parameters as *C'*.

Denote the parameters of *C *and *C' *by

$$q_{\sigma }:=\text{\# }\left\{i|u{\left(C\right)}_{i}=\sigma \right\},\;n_{\sigma }:=\text{\# }\left\{i|u{\left(C^{\prime }\right)}_{i}=\sigma \right\},$$

where *σ *∈ {-1, 0, 1}. Also let

$$n_{\sigma r}:=\text{\# }\left\{i|u{\left(C\right)}_{i}=\sigma ,u{\left(C^{\prime }\right)}_{i}=r\right\}$$

for *σ, τ *∈ {-1, 0, 1}, corresponding to the nine ways in which the classifications *C *and *C' *can overlap. This gives us the 3 × 3 contingency table for the joint classification (*C, C'*) shown in Table 2. (For notational convenience we write {-, 0, +} instead of {-1, 0, 1} when indexing variables.)

**Table 2:** Contingency table comparing predicted and experimental classifications

*n*_++_	*n*_+-_	*n*_+0_	*q*_+_
*n*_-+_	*n*_--_	*n*_-0_	*q*_-_
*n*_0+_	*n*_0-_	*n*_00_	*q*_0_
*n*_+_	*n*_-_	*n*_0_	$\left|T\right|$

Contingency table of predicted and experimental classifications. The columns sum to *n*_+_, *n*_-_, and *n*_0_, the numbers of predicted classifications of each type, and the rows sum to *q*_+_, *q*_-_, and *q*_0_, the numbers of experimental classifications of each type.

The same 3 × 3 contingency table will arise from a large number of randomized classifications $C_{R}^{\prime }$, and the number of such classifications, which we denote by *D*[*n*_++_, *n*_+-_, *n*_-+_, *n*_--_], depends only on the top left 2 × 2 corner of the table since the other entries are determined by the constraints on row and column sums. Using multinomial coefficients, we can write

$$\begin{array}{c} D\left[n_{++},n_{+-},n_{-+,}n_{--}\right]=\\ \;\left(\begin{array}{c} q_{+}\\ n_{++},n_{+-},n_{+0} \end{array}\right)\left(\begin{array}{c} q_{-}\\ n_{-+},n_{--},n_{-0} \end{array}\right)\left(\begin{array}{c} q_{0}\\ n_{0+},n_{0-},n_{00} \end{array}\right). \end{array}$$

We will write *D*[*n*_±±_] as shorthand for this quantity.

The score for a classification $C_{R}^{\prime }$ yielding this table is simply

$$S\left[n_{++},n_{+-},n_{-+},n_{--}\right]:=n_{++}+n_{--}-n_{+-}-n_{-+}.$$

We also know that the total number of possible randomized classifications is

$$D_{\text{tot}}:=\underset{n_{++},n_{+-},n_{-+},n_{--}}{\sum }D\left[n_{\pm \pm }\right]=\left(\begin{array}{c} \left|T\right|\\ n_{+},n_{-},n_{0} \end{array}\right).$$

Thus, the distribution we are seeking is a sum of the *D*[*n*_++_, *n*_+-_, *n*_-+_, *n*_--_] aggregated by the score *S*[*n*_++_, *n*_+-_, *n*_-+_, *n*_--_] and normalized by *D*_tot_. Explicitly, the probability of a score *S *is given by

$$p\left(S\right)=\underset{\left(n_{++}+n_{+-}\right)-\left(n_{-+}+n_{--}\right)=S}{\sum }\frac{D\left[n_{\pm \pm }\right]}{D_{\text{tot}}},$$

and the p-value of a score can be computed by summing the right tail of the distribution.

In the context of our illustrative example, these are the p-values given for hypotheses of interest in the "Ternary Dot Product *p*" column of Table 1. Computing these p-values naïvely is computationally intensive, however; to perform the calculations efficiently, we developed and applied an algorithm we now describe.

#### Algorithm

The Ternary Dot Product Distribution problem can be solved by computing each *D*-value individually in constant time (see Methods), giving a total running time that scales as the product *n*_+_, *n*_-_, *q*_+_, *q*_-_, i.e., *O*(*N*^4^) where *N *:= max(*n*_+_, *n*_-_, *q*_+_, *q*_-_). While this complexity is acceptable for moderate values of *N *(say up to 100), it becomes prohibitively slow for larger values of *N*, typically between 100 and 1000, that often arise in applications. Hence, faster alternatives are necessary; we give two improvements below.

Instead of computing all the *D*-values individually, we can aggregate them by the value of *n*_++ _+ *n*_--_. This still makes it possible to group them by the score *S*, as *S *only depends on *n*_++ _+ *n*_-- _and *n*_-+ _+ n_+-_. We can write the sum of all the *D*-values with a fixed *n *:= *n*_+- _+ *n*_-+ _in the form of a constant times

$$F\left[n\right]:=\underset{k}{\sum }\left(\begin{array}{c} n\\ k \end{array}\right)\left(\begin{array}{c} v-n\\ w-k \end{array}\right)\left(\begin{array}{c} x-n\\ y-k \end{array}\right),$$

where *k *= *n*_+-_, *v *= *q*_+ _+ *q*_- _- *n*_++ _- *n*_--_, *w *= *q*_+ _- *n*_++_, *x *= *n*_+ _+ *n*_- _- *n*_++ _- *n*_--_, and *y *= *n*_- _- *n*_--_. It turns out that *F*[*n*] satisfies a three-term linear recursion obtained by using the WZ algorithm [26]. With this recursion, each *F*[*n*] can be computed in average constant time. Since there are only *O*(*N*^3^) values of *F*[*n*] to compute, we get a *O*(*N*^3^) algorithm for our problem. (See Methods for a full description.)

This cubic algorithm is of theoretical interest but in practice requires exact arithmetic to obtain correct answers due to numerical instability (see Testing). We therefore developed a second algorithm that is both fast and practical, having the important advantage of working in floating-point arithmetic.

The key observation underlying our algorithm is that the vast majority of contingency tables are highly improbable (i.e., *D*[*n*_++_, *n*_+-_, *n*_-+_, *n*_--_]/*D*_tot _≪ 1) and thus may be safely ignored if we:

(a) need only carry out the computation to fixed precision; and

(b) do not care about the precise values of tail probabilities: it is enough to know that they are small.

Moreover, the quantities *D*[*n*_±±_] follow an easily described law on certain families of contingency tables, thus allowing us to identify entire families of tables that can be discarded after a constant amount of computation.

Consider families of configurations in which the row and column sums of the upper-left 2 × 2 submatrix (*n*_±±_) are fixed. Denote these sums by *r*_+_, *r*_-_, *c*_+_, *c*_-_, noting that as before, one constraint is redundant as *r*_+ _+ *r*_- _= *c*_+ _+ *c*_- _=: *t *is the total of the entries in the submatrix. Thus, in each family, one degree of freedom remains, which we may parameterize by the value of *n*_++_. It turns out that within each such family, *D*[*n*_±±_] is maximized when *n*_±± _are distributed in proportion to the 2 × 2 row and column sums, i.e.,

$$n_{\sigma \tau }\approx \tau_{\sigma }c_{\tau }/t\;\text{for}\;\sigma ,\tau \in \left\{+,-\right\}$$

(with appropriate rounding), and moreover, the probability decreases monotonically as *n*_++ _is varied in either direction from the optimum. (See Methods for details and a proof.)

**Figure 3:**
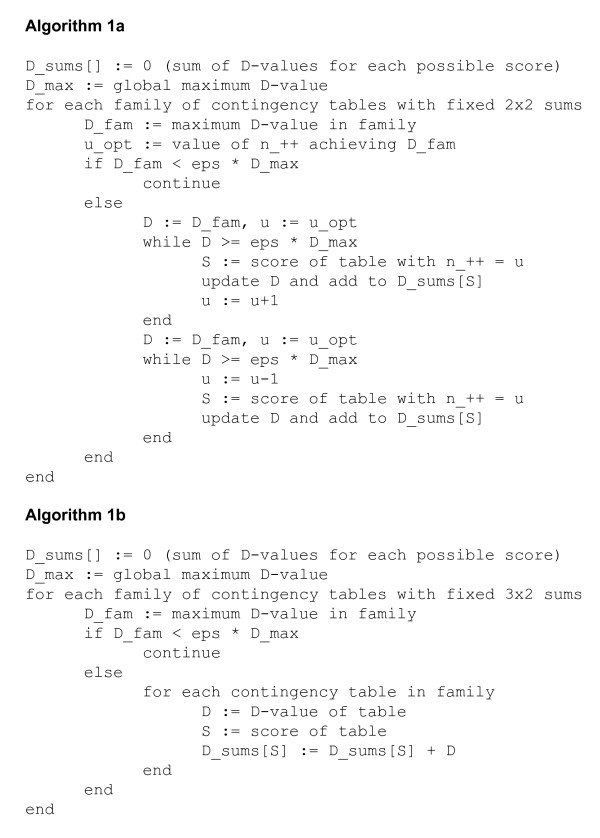
**Pseudocode for Ternary Dot Product algorithms**. Pseudocode for algorithms computing the Ternary Dot Product Distribution using thresholding on families of contingency tables.

Our algorithm thus proceeds as follows (Figure 3, Algorithm 1a). First, compute the global maximum *D*-value *D*_max _over all 3 × 3 contingency tables with row and column sums *q*_*σ*_, *n*_*τ*_. As in the 2 × 2 case just discussed, *D*_max _is achieved when $n_{\sigma \tau }\approx q_{\sigma }n_{\tau }/\left|T\right|$ for *σ, τ *∈ {+, -, 0}. Now iterate through the *O*(*N*^3^) families of contingency tables with fixed upper-left 2 × 2 row and column sums *r*_*σ*_, *c*_*τ*_. For each such family, compute its maximum *D*-value *D*_fam _by setting *n*_*στ *_≈ *r*_*σ*_*c*_*τ*_/*t *for *σ, τ *∈ {+, -} (and inferring the remaining five *n*_*στ *_with *σ *= 0 or *τ *= 0). If *D*_fam _is less than *D*_max _times a chosen threshold factor *ϵ *(perhaps machine epsilon--i.e., the maximum relative error of rounding in floating point arithmetic--divided by *N*^3^, though machine epsilon itself is likely sufficient for practical purposes), discard this family and proceed to the next one. Otherwise, the maximum probability for the family is non-negligible; in this case, iterate through the family upward and downward from the maximizing *n*_++_, updating the aggregate probabilities of the scores *S*[*n*_++_, *n*_+-_, *n*_-+_, *n*_--_] obtained, until the *D*-value of the current contingency table drops below *ϵD*_max_.

In practice, very few 2 × 2 families are within threshold. In fact, the computation time is often governed by the *O*(*N*^3^) initial threshold tests for each family (with fewer than *N*^3 ^additional *D*-value computations). This observation allows us to obtain further speedup by considering superfamilies in which only the row sums *r*_*σ *_of the upper-left 2 × 2 submatrix are fixed, leaving two degrees of freedom. Each such superfamily is the union of a set of families we considered above, and as before, the maximal *D*-value achieved by any contingency table within the superfamily is obtained by assigning counts to the left 3 × 2 submatrix proportionally to its row and column sums. We can thus apply the algorithm described above to the *O*(*N*) families of 3 × 2 left submatrices with fixed row sums. When the maximal *D*-value of the 3 × 2 family is below threshold, we may eliminate an entire one-parameter family of 2 × 2 families, achieving further efficiency (Figure 3, Algorithm 1b).

#### Testing

We tested our algorithms on a wide range of problem parameters and found that our thresholded algorithm achieves substantial speed gains across parameter distributions. Table 3 compares the scaling of run times of the simple quartic algorithm (computing all *D*-values) and Algorithm 1b, the version thresholded on 3 × 2 families, for a parameter distribution representative of typical use cases. For large cases, the thresholded algorithm reduces run times from days to minutes.

**Table 3:** Run times for Ternary Dot Product Distribution algorithm

**Problem size (*n***_**+**_**)**	Quartic algorithm:compute all *D*-values	Thresholded algorithm
8	0.05 *s*	0.07 s
16	0.19 *s*	0.15 s
32	0.92 *s*	0.36 s
64	6.16 *s*	0.61 s
128	53.15 *s*	2.35 s
256	689.18 *s*	5.93 s
512	7864.20 *s*	19.54 s
1024	> 1 d	85.76 s

Run time comparison of simple quartic Ternary Dot Product Distribution algorithm to thresholded version for an increasing family of problems with (*n*_+_, *n*_-_, *n*_0_, *q*_+_, *q*_-_) in the ratio (1, 1, 50, 2, 1), a typical usage scenario. Runs were performed on a 3.0 GHz Intel Xeon processor with 2 MB cache.

**Figure 4:**
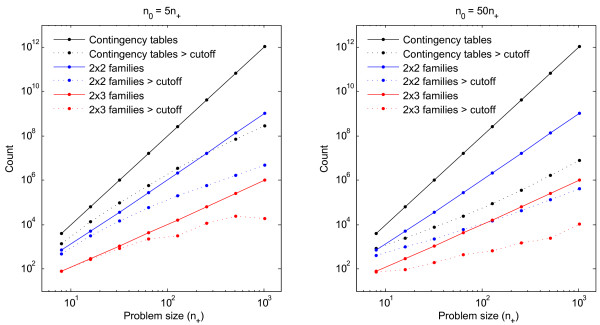
**Computational complexity of Ternary Dot Product algorithms**. Counts of the numbers of *D*-values computed by the simple quartic algorithm and during the thresholding part of the 2 × 2- and 3 × 2-family algorithms. Solid lines indicate total counts while corresponding dotted Lines indicate the numbers of contingency tables (respectively families) that pass the *ϵD*_max _threshold. The left panel shows a "dense" case *n*_0 _= 5*n*_+ _while the right panel shows a "sparse" case *n*_0 _= 50*n*_+_. For these examples we set *n*_+ _= *n*_- _= *q*_+ _= *q*_- _and chose *ϵ *= 10^-16^.

To further investigate the efficiency attained by thresholding, we computed counts of the numbers of *D*-values computed by the quartic algorithm and during 2 × 2 and 3 × 2 thresholding; we compare these counts to the actual numbers of contingency tables and families that pass threshold (Figure 4). We performed these computations for two parameter distributions: one with *n*_0 _= 5*n*_+ _and one with *n*_0 _= 50*n*_+_. The first case is relatively dense, i.e., a sizeable portion (around 30%) of the gene transcripts are significantly upregulated or downregulated. The second case is sparser; here, there are many more genes but only a few percent of them are found to be regulated. This latter case is typical in practice.

The solid black curve in Figure 4 indicates the amount of work performed by the simple quartic algorithm while the dotted black curve indicates the number of *D*-values that exceed *ϵD*_max_, thus placing a lower bound on the amount of work that any thresholding-based algorithm must perform. The disparity between these two curves immediately demonstrates the reason our thresholding algorithms achieve speedup: only a tiny fraction of the *D-*values are non-negligible. The comparison between the left and right panels of Figure 4 also makes clear the relative effects of 2 × 2 versus 3 × 2 thresholding in different parameter settings. In the dense case *n*_0 _= 5*n*_+_, we see that 2 × 2 thresholding (Algorithm 1a) is probably already close to optimally efficient: the amount of work required to do the threshold checks (solid blue curve) is comparable to the total amount of work required to compute all relevant *D*-values (dotted black line). On the other hand, in the sparse case *n*_0 _= 50*n*_+_, even performing 2 × 2 threshold checks leaves much room for improvement because the number of relevant *D*-values is far smaller. In this situation it is much more efficient to only compute *O*(*N*^2^) 3 × 2 threshold checks (solid red line). For an analytical discussion of these phenomena and a proof that the 2 × 2 thresholding algorithm has complexity *O*(*N*^3.5^), see Methods.

We have left our cubic algorithm out of the previous figures and discussion because unfortunately, our tests showed that it is numerically unstable, at least in the form stated; we now briefly discuss this issue. While the cubic algorithm does yield the correct distribution when implemented in arbitrary-precision exact arithmetic, it fails when implemented in floating-point arithmetic because the range of values in the recurrence *F*[*n*] is extremely large and subject to cancelation error. For instance, when the parameters are set to the relatively small values *v *= 20, *w *= 10, *x *= 10, *y *= 5, the values of *F*[*n*] already go from 46558512 for *n *= 0 to 6006 for *n *= 15, which means that each term is approximately a factor of 2 smaller than the previous one. We consider some alternatives in Discussion.

#### Implementation

We implemented all of our algorithms in R [27], vectorizing computations when possible. A few remarks are in order about implementation details necessary to make the thresholding algorithm numerically stable. The large factorials in the *D*-value formula require us to perform all computations in log-transformed space so as to stay within floating point range. This causes no difficulty; multiplication simply becomes addition and addition can be implemented by exponentiating the difference of two log-transformed values, adding 1, taking the log, and adding a shift. Numerically, there is no risk of cancelation error because *D*-values are only summed and never subtracted; thus, all rounding error is additive and well-controlled. The number of summands per score value *S *is *O*(*N*^3^), and using a stochastic model of rounding error, the total accumulated relative error is thus bounded by *O*(*N*^3/2^) times machine epsilon. In practice *N *is typically not more than 1000 while machine precision is 10^-16 ^so there is no concern.

The only caveat, as we noted initially, is that our algorithm guarantees precision relative to the maximum probability of all score values--not the probability of each particular score. In other words, very small tail probabilities are known only to the extent that they are understood to be negligible compared to probabilities from the bulk distribution; their precise values are not computed.

### Causal Graph Randomization

We now turn to our second computational problem arising from statistical significance evaluation in causal graph models, that of graph randomization. We begin by defining the Causal Graph Randomization problem and placing it in context with previous work on graph randomization. We then explain the special challenges of randomizing a signed causal graph and present an algorithm that successfully overcomes these challenges in practice.

#### Problem definition

The basic statistical significance question motivating our study of graph randomization is the same as before: How likely is a given observation to have occurred by chance? In the preceding development we analyzed this question from the standpoint of randomizing the identities of gene transcripts classified as upregulated or downregulated in a gene expression assay; now we take the perspective of randomizing the causal graph itself. Note that the ability to efficiently sample randomized versions of the graph allows one to create an empirical distribution of any quantitative graph property of interest, in particular enabling p-value computation.

In our setup, we estimate the p-value of a hypothesis as the proportion of the randomized graphs with a better score for the hypothesis than the actual causal graph. This is the general context in which we computed the p-values listed in the "Causal Graph *p*" column of Table 1 for our illustrative example. The precise randomization procedure involves some subtleties both in definition and algorithmic implementation, however, which we now describe.

In order to obtain an appropriate null distribution on causal graphs, it is important to require that the randomized graphs share basic structural properties with the original causal graph, yet have enough flexibility to reflect the space of reasonable graphical models. We propose to fix the vertex set *V *of our original graph *G *= (*V, E*) and randomize the edges, requiring that the randomized versions *G' *= (*V, E'*) maintain three properties:

1. Vertex degrees. We require that each vertex *a *∈ *V *have the same positive and negative in- and out-degrees in *G' *as in *G*. This requirement is important as biological networks typically have long-tailed degree distributions that include highly connected "hubs" as well as vertices with few incident edges.

2. Simplicity. We disallow self-edges and parallel edges in *G' *as these are not present in *G*. In other words, for any two vertices *a, b *∈ *V*, there cannot be an edge from *a *to itself and there can be at most one directed edge from *a *to *b*, either positive or negative.

3. Connectedness. We require that *G' *be connected, as is the case for our original biological network *G*. For our signed directed graphs, we take connectedness to mean that the graph induced by ignoring edge signs and directions is connected.

Note that the first two properties are local and the third is global. These properties capture the most significant features of a causal graph and have also been the subject of previous study in the graph randomization literature [9-12,14-16], though not until recently in the signed directed case [13,17] that we investigate here.

#### Challenges in causal graphs

In the case of undirected graphs, the randomization problem is typically solved by defining a Markov chain whose state space is *F*(*G*), the family of possible randomizations *G' *of *G*. Transitions in this chain consist of *edge switches*, which consist of picking two random edges (*a, b*) and (*c, d*) and replacing them with the edges (*a, d*) and (*c, b*), provided this does not violate required graph properties. This elementary operation yields an ergodic Markov chain whose unique stationary distribution is the uniform distribution on *F*(*G*) [9,10,13]. In the directed setting, edge switches are no longer sufficient to make the Markov chain ergodic, but adding a further operation, which we call *triangle flipping*, overcomes this problem at least for the case in which Property 3 (connectedness) is not required [12]. A triangle flip replaces the edges (*a, b*), (*b, c*), (*c, a*) (a directed 3-cycle) with the edges (*a, c*), (*c, b*), (*b, a*) (the reversed 3-cycle).

**Figure 5:**
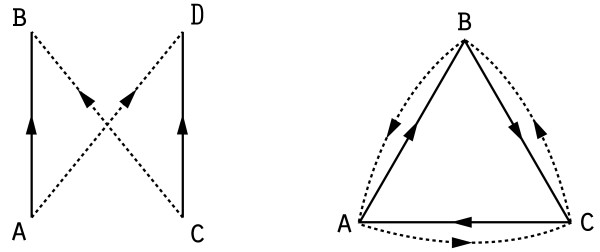
**Two obstacles to randomization of signed directed graphs**. A strong quadrilateral and a strong triangle. Solid lines indicate positive edges and dotted lines indicate negative edges.

In our situation, signed directed graphs, a natural generalization of the above randomization algorithm is to allow edge switches and triangle flips of same-sign edges. Such operations clearly preserve in- and out-degrees while modifying the edge structure of the graph, but unfortunately the sign requirement substantially constrains the set of possible transitions. We have identified several obstacles that can make parts of the state space *F*(*G*) unreachable by this method; we illustrate two in Figure 5.

The first one is the *strong quadrilateral*: a pair of edges (*a, b*), (*c, d*) of the same sign (say, +) such that the graph also contains edges (*a, d*), (*c, b*) of the opposite sign (-). The graph obtained by flipping the signs on the edges of a strong quadrilateral belongs to *F*(*G*)--indeed, it could be obtained by simultaneously performing edge switches on both pairs of edges--but neither edge switch is legal on its own because performing one edge switch would cause the pairs of edges to overlap, destroying simplicity.

The second obstacle is the *strong triangle*: a triplet of edges (*a, b*), (*b, c*), (*c, a*) of the same sign (say, +) such that the edges (*a, c*), (*c, b*), (*b, a*) of the opposite sign (-) also exist in the graph. Again, the graph obtained by flipping the signs on all the edges of a strong triangle has the same degree sequence as the original one, and it can be reached by a pair of simultaneous triangle flips, but either flip is illegal on its own. We have also found other obstacles that can be created by combinations of edge pairs, triangles and 3-paths (paths of length 3) with different signs.

Now, while these examples show that in general it is impossible to produce all the graphs in *F*(*G*) via same-sign edge switches and triangle flips, we believe that the situation is not so bleak for the large, sparse causal graphs we deal with in practice. By leveraging *auxiliary edges*, it is usually possible to bypass the above obstacles. We give one possible construction showing that strong triangles do not actually present obstacles in a large, sparse causal graph; a similar construction works for strong quadrilaterals, as well as other obstacles.

**Figure 6:**
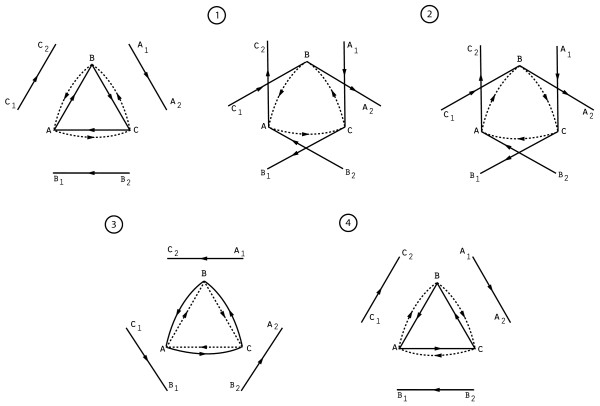
**Flipping a strong triangle using auxiliary edges**. The sequence of same-sign edge switches and triangle flips that flips a strong triangle: (1) Opening, (2) Flipping, (3) Closing, and (4) Restoring. Solid lines indicate positive edges and dotted lines indicate negative edges.

Let *a, b, c *be the vertices of a strong triangle in which (*a, b*), (*b, c*), (*c, a*) are positive edges. Suppose that there exist positive edges (*a*_1_, *a*_2_), (*b*_1_, *b*_2_), (*c*_1_, *c*_2_) disjoint from each other and {*a, b, c*}. The following procedure, illustrated in Figure 6, "flips" both parts of the strong triangle:

1. Opening: Switch (*a, b*) with (*c*_1_, *c*_2_), (*b, c*) with (*a*_1_, *a*_2_), (*c, a*) with (*b*_1_, *b*_2_).

2. Flipping: Flip the triangle (*a, c*), (*c, b*), (*b, a*) (which can now be done).

3. Closing: Switch (*a*_1_, *c*) with (*a, c*_2_), (*b*_1_, *a*) with (*b, a*_2_), (*c*_1_, *b*) with (*c, b*_2_).

4. Restoring: Switch (*b*_1_, *a*_2_) with (*c*_1_, *b*_2_) and then switch (*c*_1_, *a*_2_) with (*a*_1_, *c*_2_).

#### Algorithm

Given that causal graphs arising from biological networks are typically large and sparse, we expect that in practice the combination of same-sign edge flips and triangle switches suffices to overcome local obstacles to randomization, as observed above.

We thus propose the following algorithm for Causal Graph Randomization. Repeatedly perform the following procedure:

1. Pick two edges uniformly at random from the edge set *E*. If the edges are of different sign, restart.

2. If the edges share no endpoints, perform an edge switch if it is legal; otherwise, restart.

3. If the edges share one endpoint and belong to a directed triangle, perform a triangle flip if it is legal; otherwise, restart.

Note that in order for a transition to be legal, connectedness must be preserved (Property 3), which is a global property and thus slow to verify. To improve the efficiency of our algorithm, we therefore perform multiple iterations in between connectivity checks. We allow the number of iterations *K *between checks to vary dynamically, adopting a heuristic from Viger and Latapy [11]. More precisely, when we perform a connectivity check after *K *iterations, we proceed as follows. If the check succeeds, we multiply *K *by a factor of 1 + *Q*_+_. If it fails, we multiply it by 1 - *Q*_ and revert to the previous state of the graph (saved after the previous connectivity check *K *iterations ago). The constants *Q*_+ _and *Q*_ are chosen to match the heuristic argument presented by Viger and Latapy [11].

An important final detail of the algorithm is the number of iterations to perform; this relates to the mixing time of the Markov chain. While the mixing times of chains arising from graph randomization are not theoretically known, a constant multiple *γ *of the number of edges in the graph is enough in practice. We set *γ *= 100 by default as suggested in previous literature [14]; our tests below indicate that this value is sufficient and in fact smaller values may already suffice.

#### Testing

We tested our algorithm on the causal graph studied in our companion paper [5], which has 36,924 vertices and 248,709 edges (of which 165,037 are positive and 83,672 are negative) for an average vertex degree less than 7. To check that our randomization algorithm indeed explores the state space of possible graphs--i.e., the Markov chain mixes sufficiently--we performed 100 independent runs of the algorithm using varying numbers of iterations (corresponding to *γ *= 1, 2, ..., 100) and compared the numbers of edges shared between pairs of graphs produced at consecutive values of *γ*. The number of shared edges converged rapidly to a limiting value of ~10,200 edges in common, and in fact convergence already appeared to have happened by *γ *= 5.

We also tabulated some statistics from an independent set of 79 runs with *γ *= 100 that illuminate the workings of our algorithm. In Table 4, we give occurrence rates of local structures--in particular, potential obstacles--that our algorithm identified. We see that in our application, all of these structures were extremely rare, with strong quadrilaterals appearing only a few times per ten thousand iterations and strong triangles a few times per billion. These statistics demonstrate that local obstacles are unlikely to cause difficulty in practice.

**Table 4:** Statistics from runs of Causal Graph Randomization algorithm

Structure	Occurrence rate
Strong quadrilateral	3.76 × 10^-4^
Flippable triangle	1.22 × 10^-6^
Strong triangle	2.44 × 10^-9^

Rates of occurrence of local graph structures in 79 runs of the randomization algorithm on our test graph. A total of 5.3 billion iterations were performed during these runs.

Finally, we recorded the variation of the connectivity check interval *K *in our runs and found that on average 1163 moves were performed between checks, representing a great speedup over testing connectivity after every iteration. Even with this speedup, creating one randomized version of the graph took roughly one hour on a standard PC, a nontrivial computational cost. Note, however, that for inference on a fixed causal graph, randomized versions of the graph can be precomputed once and then used for assessing statistical significance on any number of experimental datasets.

#### Implementation

We implemented our algorithm in R using the igraph package [28]. The parameters we chose were *K *= 50 for the initial number of iterations between connectivity checks and *Q*_+ _≈ 0.131, *Q*_ ≈ 0.076 for the dynamic update of *K*. For our tests, we used a computational grid to perform independent runs of our algorithm.

## Discussion

Our work provides practical algorithms for assessing statistical significance in causal graphs but also raises a number of unresolved theoretical questions; we describe a few of them now.

In the Ternary Dot Product Distribution problem, we saw that the recursion used to obtain a cubic algorithm leads to cancelation of large approximately equal numbers. This naturally brings up the following question: Is numerical instability an artifact of a poor setup of the recursion computing *F*[*n*] or is it an inherent feature of the problem? We believe that the numerical instability is indeed an inherent feature of the problem, but it is conceivable that a clever transformation could improve the conditioning.

Another open question is the precise computational complexity of our thresholding algorithm. In Methods we prove an *O*(*N*^3.5^) bound on the complexity, but our empirical results (Figure 4) indicate that the actual performance is much faster. Can our analysis be tightened to bring down the exponent? In particular, what is the number of terms *D*[*n*_±±_] that are within a multiplicative factor of *ϵ *from the largest term *D*_max_, as a function of *N *and *ϵ*?

Furthermore, it would be interesting to investigate the consequences of level stratification in regulatory networks in order to propose a more refined null model. While such a multilevel model may indeed provide more precise estimates of statistical significance, it would be much more challenging to estimate that significance and would likely require simulation rather than an analytic approach like the one in this paper.

In the Causal Graph Randomization problem, we saw that same-sign edge switches and triangle flips are insufficient to reach all possible random graphs in the state space *F*(*G*). Does there exist an augmented set of moves that suffices? It is worth noting that (to the best of our knowledge) this question is open even in the unsigned directed case when connectedness (Property 3) is required. While edge switches and triangle flips solve the directed case without connectedness [12], these two operations do not suffice when connectedness is imposed. Indeed, consider a directed graph *G *with vertices *a, b, c, d *and directed edges (*a, b*), (*b, c*), (*c, d*). There are no triangles to flip, and the unique allowed edge switch, involving (*a, b*) and (*c, d*), disconnects the graph. Thus, in order to get to the other graph in *F*(*G*), namely, the graph with edges (*a, c*), (*c, b*), (*b, d*), a further operation, called a *3-swap *[15], is required. It is interesting to note that the triangle flip is a special case of the 3-swap where *a *= *d*.

On the other hand, in practical cases with large, sparse graphs, we showed that it is often possible to overcome local obstacles to randomization. This gives rise to the following question: Is there a lower bound on the size or upper bound on the edge density of the graph that would make same-sign edge switches and triangle flips sufficient?

An alternative approach to overcoming obstacles is to limit ourselves to edge switches and triangle flips, but allow several moves to be performed in sequence before the simplicity of the resulting graph is verified. Let *K*_*s*_(*n*) denote the longest such sequence that is required to make the resulting Markov chain on *F*(*G*) connected, where *n *is the number of vertices of *G*. It is clear that *K*_*s*_(*n*) is always finite and in fact bounded by *n*^2 ^-- *n*, the largest number of edges in a simple graph on *n *vertices. Does *K*_*s*_(*n*) grow linearly with *n*, is it bounded above by a constant, or something in between?

Finally, even in cases that Markov chains can be shown to generate all possible graph randomizations, their mixing time remains an open question. It is known that the Markov chain rapidly mixes in the case of *regular *directed graphs, i.e., graphs in which all vertices have the same in- and out-degrees [16], but it appears to be slowly mixing for some exponential degree distributions [29]. It would be interesting to better understand the mixing time behavior of the chain we proposed for signed directed graphs.

In some cases it may be possible to reduce the size of a causal graph, and thereby the resources required to solve the Causal Graph Randomization problem, by performing a transitive reduction of the graph. A transitive reduction of a graph is a minimal graph with the same transitive closure as the original graph (so a transitive reduction does not contain any edges between vertices that are connected by a different path in the graph). Transitive reduction has been successfully used in computational biology [30]; we opted not to use it here to avoid the possibility of filtering out potentially useful relationships, particularly because our graph likely contains some noise. This reduction approach might prove most helpful when some causal relationships in the graph are known *a priori *to be indirect.

## Conclusions

This paper presents the first systematic attempt at addressing the computational challenges that arise in the evaluation of the significance of results produced by a causal graph-based model. We develop two algorithms for the Ternary Dot Product Distribution problem and one algorithm for the Causal Graph Randomization problem. All the algorithms are implemented in the statistical computing language R and available on request for academic purposes. We believe that our work opens the door to further study of causal graphs from both a theoretical and practical perspective, and we hope that these algorithms will enable the integration of statistical significance computations into causal graph-related methods in biology and other areas of science.

## Methods

### Quartic algorithm for Ternary Dot Product Distribution

The Ternary Dot Product Distribution problem can be solved with a simple algorithm using the following relationships between the *D*-values:

$$\begin{array}{c} \frac{D\left[i,j,k,l+1\right]}{D\left[i,j,k,l\right]}=\frac{q_{-}-\left(j+l\right)}{l+1}\cdot \frac{n_{-}-\left(k+l\right)}{r+\left(i+j+k+l\right)};\\ \frac{D\left[i,j,k+1,l\right]}{D\left[i,j,k,l\right]}=\frac{q_{+}-\left(i+k\right)}{k+1}\cdot \frac{n_{-}-\left(k+l\right)}{r+\left(i+j+k+l\right)};\\ \frac{D\left[i,j+1,k,l\right]}{D\left[i,j,k,l\right]}=\frac{q_{-}-\left(j+l\right)}{j+1}\cdot \frac{n_{+}-\left(i+j\right)}{r+\left(i+j+k+l\right)};\\ \frac{D\left[i+1,j,k,l\right]}{D\left[i,j,k,l\right]}=\frac{q_{+}-\left(i+k\right)}{i+1}\cdot \frac{n_{+}-\left(i+j\right)}{r+\left(i+j+k+l\right)}; \end{array}$$

where $r:=q_{0}+n_{0}-\left|T\right|+1$.

This algorithm can be made numerically stable by computing an initial normalized value *D*[0, 0, 0, 0]/*T*, so that all the values throughout the recurrence stay between 0 and 1. (There is a slight subtlety that if *r *≤ 0 we need to use an initial value other than (0, 0, 0, 0).)

### Cubic algorithm for Ternary Dot Product Distribution

Setting *γ*_1 _:= *n*_++ _+ *n*_--_, *γ*_2 _:= *n*_-+ _+ *n*_+-_, *s *:= *n*_++_, *t *:= *n*_+-_, we rewrite *D*[*n*_±±_] as

$$\begin{array}{c} \left(\begin{array}{c} q_{+}\\ s,t,r_{+} \end{array}\right)\left(\begin{array}{c} q_{-}\\ \gamma_{1}-s,\gamma_{2}-t,r_{-} \end{array}\right)\\ \;\cdot \left(\begin{array}{c} q_{0}\\ n_{-}-\gamma_{1}+\left(s-t\right),n_{+}-\gamma_{2}-\left(s-t\right),r_{0} \end{array}\right), \end{array}$$

where *r*_+ _:= *q*_+ _- (*s *+ *t*), *r*_- _:= *q*_- _- (*γ*_1 _+ *γ*_2_) + (*s *+ *t*), *r*_0 _:= *r *- 1 + (*γ*_1 _+ *γ*_2_). By rearranging the factorials, we can further rewrite this expression as

$$\begin{array}{c} \frac{\left(\begin{array}{c} \gamma_{1}\\ s \end{array}\right)}{\left(\begin{array}{c} q_{+}+q_{-}\\ q_{+} \end{array}\right)}\left(\begin{array}{c} q_{+}+q_{-}\\ \gamma_{1},\gamma_{2},\left(q_{+}+q_{-}-\left(\gamma_{1}+\gamma_{2}\right)\right) \end{array}\right)\\ \;\;\;\cdot \left(\begin{array}{c} q_{0}\\ n_{+}+n_{-}-\left(\gamma_{1}+\gamma_{2}\right) \end{array}\right)f\left(\gamma_{1},\gamma_{2},s,t\right), \end{array}$$

where

$$\begin{array}{c} f\left(\gamma_{1},\gamma_{2},s,t\right):=\\ \;\left(\begin{array}{c} \gamma_{2}\\ t \end{array}\right)\left(\begin{array}{c} q_{+}+q_{-}-\left(\gamma_{1}+\gamma_{2}\right)\\ q_{+}-\left(s+t\right) \end{array}\right)\left(\begin{array}{c} n_{+}+n_{-}-\left(\gamma_{1}+\gamma_{2}\right)\\ \left(n_{-}-\gamma_{1}\right)+\left(s-t\right) \end{array}\right). \end{array}$$

Note that the product above only depends on *t *through *f*(*γ*_1_, *γ*_2_, *s, t*). If we could compute *F*(*γ*_1_, *γ*_2_, *s*) := ∑_*t *_*f*(*γ*_1_, *γ*_2_, *s, t*) in constant time per term, we would obtain a cubic algorithm instead of a quartic one.

Let us now define

$$F\left[n\right]:=\underset{k}{\sum }\left(\begin{array}{c} n\\ k \end{array}\right)\left(\begin{array}{c} v-n\\ w-k \end{array}\right)\left(\begin{array}{c} x-n\\ y-k \end{array}\right),$$

where we made the following substitutions to simplify the previous expression: *n *:= *γ*_2_, *k *:= *t, v *:= *q*_+ _+ *q*_- _- *γ*_1_, *w *:= *q*_+ _- *s, x *:= *n*_+ _+ *n*_- _- *γ*_1_, *y *:= *n*_- _- *γ*_1 _+ *s*.

By using the WZ algorithm [26], we obtain the following recursion on *F*[*n*]:

$$\begin{array}{c} \left(n+2\right)\left(n+1\right)a_{0}F\left[n\right]\\ -\left(n+2\right)\left(b_{0}+b_{1}n+b_{2}n^{2}+b_{3}n^{3}+b_{4}n^{4}\right)F\left[n+1\right]\\ +\left(n-x+1\right)\left(n-v+1\right)\\ \;\;\cdot \left(c_{0}+c_{1}n+c_{2}n^{2}+c_{3}n^{3}\right)F\left[n+2\right]\\ -d_{3}F\left[n+3\right]=0, \end{array}$$

where the coefficients of the polynomial multipliers are given in Additional File 1.

### Practical algorithm for Ternary Dot Product: Mathematical details and *O*(*N*^3.5^) complexity bound

Consider families of contingency matrices in which the row and column sums of the upper-left 2 × 2 submatrix (*n*_±±_) are fixed. Denote these sums by *r*_+_, *r*_-_, *c*_+_, *c*_-_, noting that as before, one constraint is redundant as *r*_+ _+ *r*_- _= *c*_+ _+ *c*_- _=: *t *is the total of the entries in the submatrix. Thus, in each family, one degree of freedom remains, which we may parameterize by the value of *n*_++ _=: *u*.

Within each such family, the values of *n*_0+_, *n*_0-_, *n*_+0_, *n*_-0_, *n*_00 _are determined by *r*_+_, *r*_-_, *c*_+_, *c*_- _and thus independent of *u*. It follows that relative *D*-values within a family obey the simple proportionality relation

$$D\left[n_{++},n_{+-},n_{-+},n_{--}\right]\propto \frac{1}{\left(n_{++}\right)!\left(n_{+-}\right)!\left(n_{-+}\right)!\left(n_{--}\right)!}.$$

Explicitly, the proportionality constant is $\left|T\right|!/\left(n_{0+}!n_{0-}!n_{+0}!n_{-0}!n_{00}!\right)$. We now observe that the expression on the right is maximized when *n*_±± _are distributed in proportion to the 2 × 2 row and column sums, i.e.,

$$n_{\sigma \tau }\approx \tau_{\sigma }c_{\tau }/t\;\text{for}\;\sigma ,\tau \in \left\{+,-\right\}$$

(with appropriate rounding), and moreover, the probability decreases monotonically as *u *is varied in either direction from the optimum. To see this, observe that the multiplicative change Δ*D*[*u*] in *D *upon decrementing *u *is simply

$$\begin{array}{c} \frac{D\left[u-1,r_{+}-u+1,c_{+}-u+1,r_{-}-c_{+}+u-1\right]}{D\left[u,r_{+}-u,c_{+}-u,r_{-}-c_{+}+u\right]}\\ \;=\frac{u\left(r_{-}-c_{+}+u\right)}{\left(r_{+}-u+1\right)\left(c_{+}-u+1\right)}. \end{array}$$

The numerator and denominator are both monic quadratics in *u *and hence cross at precisely one point which is easily computed, giving the result claimed.

We now provide an argument that our algorithm performs no more than $O\left(\sqrt{N}\right)$ iterations per family, proving an *O*(*N*^3.5^) bound on the complexity of the overall algorithm. Denote by *u*_opt _≈ *r*_+_*c*_+_/*t *the value of *n*_++ _maximizing *D *for a given family and compare

$$\begin{array}{ll} \Delta D\left[u_{\text{opt}}\right] & =\frac{u_{\text{opt}}\left(r_{-}-c_{+}+u_{\text{opt}}\right)}{\left(r_{+}-u_{\text{opt}}+1\right)\left(c_{+}-u_{\text{opt}}+1\right)}\approx 1,\;\\ \Delta D\left[u\right] & =\frac{u\left(r_{-}-c_{+}+u\right)}{\left(r_{+}-u+1\right)\left(c_{+}-u+1\right)}.\;\\ & \; \end{array}$$

As *u *decreases from *u*_opt_, observe that the terms in the numerator of Δ*D*[*u*] each decrease in unit intervals while the terms in the denominator each increase. It follows just from restricting our attention to the first term in the numerator that

$$\Delta D\left[u_{\text{opt}}-k\right]\leq \frac{u_{\text{opt}}-k}{u_{\text{opt}}}\leq 1-\frac{k}{N}.$$

(In fact, it is not hard to see that all four terms contribute such factors, but for the purpose of asymptotics our bounds need not be tight.) Chaining these bounds together,

$$\begin{array}{ll} \frac{D\left[u_{\text{opt}}-K\right]}{D\left[u_{\text{opt}}\right]} & \leq \Delta D\left[u_{\text{opt}}-K-1\right]\cdots \Delta D\left[u_{\text{opt}}\right]\;\\ & \leq \overset{K-1}{\underset{k=1}{\prod }}\left(1-\frac{k}{N}\right),\;\\ & \; \end{array}$$

from which it follows that the *D*-value drops below *ϵ *times the family optimum within $K=O\left(\sqrt{N\text{log}\left(1/\varepsilon \right)}\right)$ iterations, or for fixed *ϵ*, $K=O\left(\sqrt{N}\right)$.

## Authors' contributions

LC and PL developed and tested the methods and drafted the manuscript. LC implemented the algorithms in R and reviewed the literature. AE prepared the illustrative example application. BB participated in the design and coordination of the project and helped draft the manuscript. DZ conceived of the project, participated in its design and coordination, and helped draft the manuscript. All authors read and approved the final manuscript.

## Supplementary Material

Additional file 1 **Recurrence relation for Ternary Dot Product Distribution cubic algorithm**. Details of recurrence relation for *F*[*n*] in cubic algorithm. Click here for file
