# Supplementary material for: Assessing statistical significance in causal graphs
Source: BMC Bioinformatics. 2012 Feb 20;13:35. doi: 10.1186/1471-2105-13-35 (PMC3307026; doi:10.1186/1471-2105-13-35)
Supplement: Additional file 1 — Recurrence relation for Ternary Dot Product Distribution cubic algorithm. Details of recurrence relation for F[n] in cubic algorithm. [file 1471-2105-13-35-S1.PDF]

Additional File 1: Recurrence relation for Ternary Dot Product Distribution cubic algorithm.

Using the notation from the main manuscript, $F[n]$ satisfies the following recurrence relation:

$$
\begin{aligned}
&-(n+2)(n+1)a_0F[n] + (n+2)(b_0 + b_1n + b_2n^2 + b_3n^3 + b_4n^4)F[n+1]- \\
&-(n-x+1)(n-v+1)(c_0 + c_1n + c_2n^2 + c_3n^3)F[n+2] + d_3F[n+3] = 0,
\end{aligned}
$$

where

$$
\begin{aligned}
a_0 &:= (n-(w+x)+y)(n-v+w-y)((n+1)z_0 + 2z_1) \\
b_0 &:= 4(3vx+1)z_1 - (7vx-2)z_2 - (3vx+1)z_3 - (4vx+7)z_5 - 2z_7 + \\
&\quad + 4x(x-1)w^2(w-y) + 2x^3(v^2+w^2) + 6vx(w^2 - y^2(v-1))+ \\
&\quad + 2v(v-1)y^2(v-2(w-y)) + (v^3+x^3) + 4(vwxy + 2vx)(v+x)- \\
&\quad - 2w^2x^2(3v+1) + 3v^3y + wx^3 - 9vx(w+y) + 2(v^2x^2)(v+5) \\
b_1 &:= 3z_0 + (18vx+24)z_1 - (vx-14)z_2 - 7z_3 + 2z_4 - 21z_5 - 6z_6 - 2z_7- \\
&\quad - 4(w-y)(2x-1)w^2 + (2v-1)y^2) - 18vx(v+x) - 4(v^2y^2 + w^2x^2)- \\
&\quad - 16vy(wx - y(w-y)) + 12vx(w^2+y^2) - 3(x^3(v-w) + v^3(x-y))+ \\
&\quad + 4wy(v+x) - 8y^2(w-y) - 31vx(w+y) + 4v^2x^2 + 2(v^3+x^3) + 43vx(v+x) \\
b_2 &:= 10z_0 + 38z_1 + 22z_2 - 11z_3 - 14z_5 - 4z_6 + 4(w^3+y^3) - 2(v-x)(w^2-y^2)- \\
&\quad - 4wy((w+y)-(v+x)) - 22(w+y)vx + v^3 + x^3 + 17vx(v+x) \\
b_3 &:= 8z_1 + 10(v+x)(w+y) - 5(v+x)^2 + 11z_0 \\
b_4 &:= 4z_0 \\
c_0 &:= 24z_1 + 4z_2 - 4(v^2y^2 + w^2x^2 - v^2x^2) - 2(v^2+x^2) + 12(x^2(v-w)+ \\
&\quad + v^2(x-y) - vx(w+y)) - 16vwxy + 8(vw(x^2+y^2) + xy(v^2+w^2)) \\
c_1 &:= 14z_0 + 44z_1 + 14z_2 - 7z_3 + 4z_4 - 8z_5 - 8((w+y)wy - (v+x)vx) - 12vx(w+y) \\
c_2 &:= 17z_0 + 16z_1 + 6z_2 - 3z_3 \\
c_3 &:= 5z_0 \\
d_3 &:= 2(n-x+1)(n-x+2)(n-v+2)(n-v+1)(nz_0 + 2z_1)
\end{aligned}
$$

with the auxiliary variables

$$
\begin{aligned}
&z_0 := (x+v) - 2(w+y); \quad && z_1 := vy + wx - vx; \quad && z_2 := vw + xy; \quad && z_3 := v^2 + x^2; \\
&z_4 := vy^2 + w^2x; && z_5 := v^2y + wx^2; && z_6 := vw^2 + xy^2; && z_7 := v^2w + x^2y.
\end{aligned}
$$
